# A New Genotype Imputation Method with Tolerance to High Missing Rate and Rare Variants

**DOI:** 10.1371/journal.pone.0101025

**Published:** 2014-06-27

**Authors:** Yumei Yang, Qishan Wang, Qiang Chen, Rongrong Liao, Xiangzhe Zhang, Hongjie Yang, Youmin Zheng, Zhiwu Zhang, Yuchun Pan

**Affiliations:** 1 School of Agriculture and Biology, Shanghai Jiao Tong University, Shanghai, China; 2 National Animal Husbandry Services, Beijing, China; 3 Institute for Genomic Diversity, Cornell University, Ithaca, New York, United States of America; 4 Shanghai Key Laboratory of Veterinary Biotechnology, Shanghai, China; 5 Department of Crop and Soil Science, Washington State University, Pullman, Washington, United States of America; University of Miami, United States of America

## Abstract

We report a novel algorithm, iBLUP, to impute missing genotypes by simultaneously and comprehensively using identity by descent and linkage disequilibrium information. The simulation studies showed that the algorithm exhibited drastically tolerance to high missing rate, especially for rare variants than other common imputation methods, e.g. BEAGLE and fastPHASE. At a missing rate of 70%, the accuracy of BEAGLE and fastPHASE dropped to 0.82 and 0.74 respectively while iBLUP retained an accuracy of 0.95. For minor allele, the accuracy of BEAGLE and fastPHASE decreased to −0.1 and 0.03, while iBLUP still had an accuracy of 0.61.We implemented the algorithm in a publicly available software package also named iBLUP. The application of iBLUP for processing real sequencing data in an outbred pig population was demonstrated.

## Introduction

Benefited from the advances of sequencing technologies, Genome-Wide Association Studies (GWAS) have revealed substantial genetic loci controlling human diseases and agriculturally important traits [1]–[3]. However, the identified loci collectively explain only a small proportion of total variation [4]–[7]. In addition to the path of common diseases and common variants, the new path of common disease and rare variants shed a new hope to have a better understand of complex traits [8]. Multiplexing is one the advances that revolutionized the high throughput Genotyping By Sequencing (GBS). Samples are individually tagged and pooled into a single lane of flow cell. It exponentially increases the number of samples analyzed in a single run without dramatically increasing cost and time [9].

Recently, several GBS methods used for both inbred and outbred population have been developed [10], [11]. The challenge is that the sequencing data contains a lot of missing genotypes. Imputation of missing genotypes at high missing rate is hard and imputation for rare variants are extreme hard, especially for general outbred populations because of the high degree of heterogeneity and phase ambiguity in the haplotype [12].

The information for imputation can be divided into two categories. One is linkage disequilibrium (LD) among genetic loci; the other is the relationship, termed identity by decent (IBD), among individuals [13]. Imputation methods have been developed to use either of them, or both with different degrees of complexity. These methods include allele frequencies based methods(PLINK, SNPMSTAT, UNPHASED, and TUNA), Hidden Markov Chain based methods(IMPUT, MACH, fastPHASE), mixed model based methods (S-MM,M-MM) and graphic theory based method(BEAGLE) [14]–[22]. A clear linkage phase, such as a haplotype, is the most desirable situation for most of the algorithms to work with [13]. However, phasing becomes extremely difficult with GBS at low coverage with high missing rate, especially in outbred population such as human, maize landrace, dog, cattle and pig where heterogeneity is high [23]. As we known, none of the existing methods can work well for the GBS data with low coverage and high missing rate in outbred population and no convenient software can impute the missing genotypes based on this kind of data. The objective of this study was to make full usage of LD and IBD simultaneously and develop a genotype imputation algorithm and software with tolerance to high missing rate, especially for rare variants.

## Methods

Approval by the Institutional Animal Care and Use Committee of Shanghai Jiao Tong University (contract no. 2011–0033) was given for all experimental procedures involving pigs in the present study. All the 72 sequenced pigs were housed in Shanghai Xiangxin Livestock Ltd. Co., Shanghai, China, and were raised according to the standard practice for housing and care of Xiangxin Livestock Ltd. Co.(http://www.shxxgx.com/sygl.htm). Additional information of the sequenced pigs was shown in **Table S1**.

### 1 iBLUP method

The chromosomes were divided into a large number of blocks on the basis of the extent of LD, and the LD of any two markers in a block is necessarily greater than some criteria. The marker that is less than some criteria will be removed from the block and will be imputed by a single variable BLUP model. All the markers in one block were analyzed by modeling using multivariate BLUP, and missing genotypes were predicted simultaneously. The imputation model for each marker in the block was:



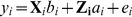
(1)Where *y_i_* is an observed genotype vector for the number of copies of the minor allele (0, 1 or 2) for the marker, The length of the vector equals the number of individuals, *b_i_* is the fixed effect and X*_i_* is the design matrix for *b_i_*, *a_i_* is the effect underlying the observed genotype, Z*_i_* is the design matrix for *a_i_* and *e_i_* is the residual. The vector *b_i_* and a*_i_* have the same length as *y_i_*.

Assumed that there are m markers in one block, i from 1 to m, we set
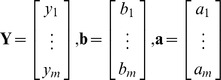


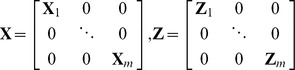



The multivariate BLUP equations are:
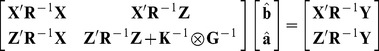
(2)where *R* is the residual variance–covariance matrix. Following Gengler et al. (2007), we set



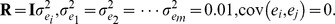



*G* is the genetic variance–covariance matrix
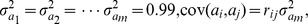
where *r_ij_* is the correlation between markers *i* and *j*. When the value of *r_ij_* was >0.95 (or <−0.95), *r_ij_* was set to 0.95 (or −0.95) to avoid the singularities matrix [22].



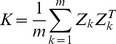
is a marker-based kinship matrix [24] and we develop an iterative kinship algorithm to construct the matrix considering the missing data described in a later section. The symbol 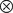 represents the Kronecker product.

### 2 Iterative kinship algorithm

In the model, *K* was calculated using the iterative algorithm because of the high missing rate of the genomic genotype data. The initial *K* was calculated using only the genotype data from genotyped individuals, and homogenized after dividing by the number of common typed loci. The following *K* was calculated using the imputed genotype based on BLUP. When the difference between the correlation coefficients of two continuous *K* values is <0.001, the iterative process is finished. To ensure that *K* converged, we forced the imputed genotype of multivariate BLUP to be 0 (or 2), if it is <0 (or >2). To improve the computation speed, the Equation (2) was solved with an LU-factored method based on subroutines from the Intel^R^ Math Kernel Library using parallel execution on Linux workstations.

### 3 iBLUP pipeline

The iBLUP pipeline can deal with both SNP array and sequencing data. The analysis steps are dependent on the kind of input data. If SNP array data is the input data, only step 5 need be performed. If user wants to impute sequencing data directly, all the 5 steps can be run automatically. We introduce the analysis steps briefly as follow, and the details can be found in the online manual (http://klab.sjtu.edu.cn/iBLUP/).

**Step 1.** To assign raw sequencing reads to individuals.

**Step 2.** To filter reads on quality.

**Step 3.** To map qualified reads to reference genome. The qualified reads are clustered by aligning reads with the reference genome using BWA [25] by the following steps. We first mapped all filtered reads to the reference panel and then attempted to divide remaining single reads into two or three shorter reads according to the sequences of enzyme cutting sites to align them individually with the reference genome because of uncertain ligation, such as adapter-DNA-DNA-adapter. We then used the sliding window method to query the remaining reads to ensure that we can make use of the incomplete reference panel. The rule of the sliding window is that the selected 25 uninterrupted bases from the first base at the 5′ end of a read was aligned with the reference genome and a single base was added at each alignment until the maximum aligned sequence was reached. If the first 25 bp at the 5′ end were not aligned successfully, the next 25 bp, i.e. base pairs 2–26, were aligned with the reference genome and so on.

**Step 4.** To call genotype for each marker and individual. Reads that aligned with the reference panel were stored as “sam” files. Our iBLUP applied SAMtools to discover SNPs [26].

**Step 5.** To impute missing genotypes by iBLUP.

### 4 Simulation data

There were 3220 individuals genotyped on 9990 markers in the 15^th^ QTL-MAS workshop [27]. The 9,990 SNP markers were evenly distributed on 5 chromosomes. Each chromosome had a size of 1 Morgan and carried 1998 SNPs equally distributed (1 SNP every 0.05 cM). The 3220 individuals were from two generations, of which 220 individuals (200 females and 20 males) are parents, and the remaining 3000 individuals are offspring to be divided into 200 full-sib families consisting of 15 progeny per dam, which were generated by regular cross-hybridization of male and female parents (**See Figure S1**a). All the genotype of 9,990 SNPs on the 3220 individuals are known. Subset of individuals were sampled from the workshop data under two sampling schema: 1) Half sib schema. The sampled data included all the parents (20 males and 200 females) and two progeny selected randomly from each full-sib family. This schema sampled more families with smaller family size (**See Figure S1**b). 2) Full sib schema. The sample data included 5 males, the corresponding mates and eleven progeny from each full sib family. This schema sampled fewer families with larger family size (**See Figure S1**c). A subset of markers were sample from the entire genetic markers (9,990) to investigate the effect of marker density. One of marker was selected for every five adjacent five markers. The sampled marker data set contained 1998 markers. The known genotype data were randomly masked as missing data at specific proportions. The proportions were ranged from 10% to 80%. Accuracy was calculated as Pearson correlation coefficient between known genotype and imputed.

### 5 Real sequencing data

The data were generated from an Illumina High-seq 2000 sequencer. A flow-cell lane was used to sequence 72 pigs (36 Landrace pigs and 36 Large White pigs) by using a DNA barcoding and genome reducing protocol (http://klab.sjtu.edu.cn/GGRS/). There were 380,971,530 raw reads. The number of reads per individual ranged from 1,570,923 to 10,077,526 and the average was 5,022,387.

## Results

**Figure 1:**
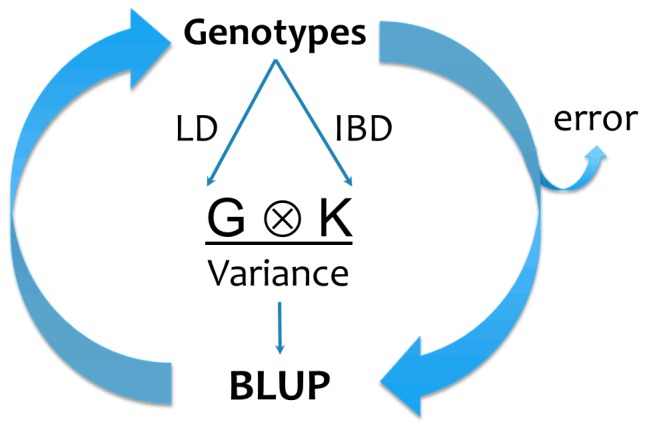
The mechanism and performance of iBLUP. The figure illustrates how observed genotype with missing value is imputed by Best Linear Unbiased Prediction (BLUP). The imputation uses both relationship among markers represented by Linkage Disequilibrium (LD), and relationship among individuals represented as Identity By Decent (IBD).G and K are the genetic variance–covariance matrix and marker-based kinship matrix respectively, and the symbol 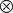 represents the Kronecker product.

We were motivated to develop a non-phasing algorithm [28] in 5a multivariate mixed model (M-MM) [22]. To take full advantage of a M-MM to fully incorporate both LD and IBD simultaneously, we made two major changes to enhance the representations of marker IBD information on the relationship matrix (K) among individuals, and marker LD information on the covariance matrix (G) of underlying variables (See **Figure 1**). First, we replaced overall IBD derived from the pedigree by the IBD derived from the markers. We developed an iterative algorithm to derive a robust IBD to situations with missing genotypes. Second, instead of using an arbitrary fixed size of LD block (e.g. two mega base pair), we implemented an optimization process to determine the LD threshold that to determine a variable size of LD block. The value of LD was represented as the squared correlation coefficient (*r* ^2^) calculated for the markers on the LD block. Our improved method of imputation by Best Linear Unbiased Prediction (iBLUP) had markedly higher accuracy than the conventional M-MM method, even higher than the sophisticated graphic phasing method (BEAGLE), especially for situations with high missing rate.

The performance of iBLUP was compared to three other types of commonly used methods, M-MM, BEAGLE and fastPHASE on a data set from 15^th^QTLMAS. The iBLUP method outperformed over M-MM at all range of missing rates. When missing genotypes were below 40%, iBLUP had similar accuracy to BEAGLE and fastPHASE. With higher missing rates, iBLUP markedly outperformed BEAGLE and fastPHASE. At a missing rate of 50%, the accuracy of fastPHASE dropped to 0.79 while iBLUP retained an accuracy of 0.98. At a missing rate of 70%, the accuracy of BEAGLE fell to 0.82 while iBLUP held an accuracy of 0.95 (**Table 1**).

**Table 1:** The comparison of four genotype imputation methods: iBLUP, BEAGLE, M-MM and fastPHASE.

Method	10%	20%	30%	40%	50%	60%	70%	80%
iBLIUP	0.99	0.99	0.99	0.99	0.98	0.97	0.95	0.92
BEAGLE	0.99	0.99	0.99	0.99	0.99	0.95	0.82	0.76
M-MM	0.90	0.89	0.89	0.87	0.87	0.85	0.82	0.71
fastPHASE	0.99	0.99	0.99	0.99	0.79	0.76	0.74	0.69

It is critical to dissect overall accuracy across all genotypes into major and minor allele genotypes. The major genotypes can be accurately imputed for rare variants if the accuracy of minor allele is ignored. The iBLUP method is superior to BEAGLE and fastPHASE not only on overall accuracy, but also for minor allele genotypes. When missing rate was 60%, the accuracy of fastPHASE dropped to −0.01, iBLUP kept an accuracy of 0.72 for minor allele genotypes. At missing rate of 70%, the accuracy of BEAGLE dropped to −0.1, while iBLUP still retained an accuracy of 0.61 for minor allele genotypes (**Table 2**).

**Table 2:** Imputation accuracy characterized by minor allele*.

		60%			70%			80%		
Genotype	MAF	iBLUP	BEAGLE	fastPHASE	iBLUP	BEAGLE	fastPHASE	iBLUP	BEAGLE	fastPHASE
Major	<5%	1.00±0.0002	1.00±0.0000	0.99±0.009	1.00±0.0001	0.99±0.0000	0.99±0.0005	1.00±0.0002	0.99±0.0000	0.99±0.0003
	5–25%	0.96±0.0005	0.94±0.0002	0.76±0.0157	0.94±0.0002	0.88±0.0001	0.73±0.0112	0.91±0.0003	0.87±0.0000	0.70±0.0081
	>25%	0.89±0.0011	0.88±0.0004	0.42±0.0311	0.85±0.0011	0.68±0.0008	0.37±0.0221	0.76±0.0009	0.64±0.001	0.30±0.014
	All	0.97±0.0003	0.96±0.0001	0.79±0.0134	0.96±0.0002	0.89±0.0002	0.77±0.0096	0.93±0.0002	0.87±0.0002	0.74±0.0063
Minor	<5%	0.10±0.0013	−0.07±0.0027	−0.04±0.0095	0.05±0.0015	−0.10±0.0008	−0.05±0.0074	0.00±0.0018	−0.11±0.0008	−0.06±0.0062
	5–25%	0.52±0.0029	0.27±0.0018	−0.04±0.0273	0.38±0.0017	−0.24±0.0011	−0.08±0.0194	0.20±0.0014	−0.30±0.0001	−0.13±0.0113
	>25%	0.77±0.0019	0.7±0.0013	0.11±0.0389	0.67±0.0018	−0.07±0.003	0.06±0.0288	0.51±0.0016	−0.41±0.0017	0.01±0.0195
	All	0.72±0.0021	0.59±0.0011	−0.01±0.0171	0.61±0.0017	−0.1±0.0024	0.03±0.026	0.44±0.0015	−0.38±0.0013	−0.01±0.0171
All		0.97±0.0003	0.95±0.0002	0.76±0.0164	0.95±0.0003	0.82±0.0004	0.74±0.0119	0.92±0.0002	0.76±0.0000	0.69±0.0079

*Genetic markers were classified into three categories based on Minor Allele Frequency (MAF). The cutoffs of MAF were 5% and 25%. Known genotypes were masked as missing at three different rates: 60%, 70% and 80%. Three imputation methods (BEAGLE, fastPHASE and iBLUP) were used to impute the masked genotypes. Accuracy was calculated as Pearson correlation coefficient between known genotype and imputed. Three subset of genotypes were examined: 1) Genotypes with major allele (Major), including homozygous of major allele and heterozygous; 2) Genotypes with minor allele (Minor), including homozygous of minor allele and heterozygous; 3) Genotypes of two homozygous and heterozygous (All).

We expanded our examination over a variety of circumstances. First we examined the responses of imputation accuracy to the level of kinship among individuals. Two subsets of the data from the QTLMAS 15th dataset were used for the examination. The two datasets contained all the available markers with the average LD value of 0.137, but varied on family structure. The first dataset consisted of a family structure of two full-sib individuals sampled from each family and the second dataset consisted of a family structure of parents and their eleven progeny. The average kinship coefficients were 0.0073 and 0.048 for the first and second family structures, respectively. In both cases, iBLUP had better imputation accuracy than BEAGLE and fastPHASE at missing rates ranged from 60% to 80% (**Table 3**).

**Table 3:** Responses of imputation accuracy on marker density and individual relationship*.

Missing	rate		60%			70%			80%	
		iBLUP	BEAGLE	fastPHASE	iBLUP	BEAGLE	fastPHASE	iBLUP	BEAGLE	fastPHASE
Sibs^a^	Half	0.97±0.0006	0.95±0.0002	0.76±0.0163	0.95±0.0005	0.82±0.0004	0.74±0.0081	0.92±0.0002	0.76±0.0002	0.69±0.0041
	Full	0.97±0.0003	0.96±0.0002	0.79±0.0113	0.96±0.0006	0.83±0.0004	0.75±0.0076	0.94±0.0007	0.77±0.0002	0.72±0.0046
Density^b^	High	0.97±0.0006	0.95±0.0002	0.76±0.0163	0.95±0.0005	0.82±0.0004	0.74±0.0081	0.92±0.0002	0.76±0.0002	0.69±0.0041
	Low	0.90±0.0006	0.85±0.0005	0.76±0.0122	0.87±0.0004	0.78±0.0003	0.73±0.0093	0.83±0.0007	0.75±0.0006	0.71±0.0062

*The full dataset from 15^th^ QTL-MAS workshop was sampled on individual relationship and marker density. The full dataset contains 3220 individuals genotyped with 9990 markers. The 3220 individual include 20 sires, 200 dams (10 dam per sire), and 3000 progeny (15 progeny per dam) as displayed in **Figure S1a**. The full population were randomly sampled to form two sub populations, one with individuals more related each other (full sibs see **Figure S1c**) and the other with individuals less related each other (half sibs, see **Figure S1b**). The known genotypes were randomly masked as missing at three different rates: 60%, 70%, and 80%. Two imputation methods (BEAGLE, fastPHASE and iBLUP) were used to impute the masked genotypes. Accuracy was calculated as Pearson correlation coefficient between known genotype and imputed. The sampling of missing genotypes was repeated ten times. The average and standard error of imputation accuracy are reported in the table.

a All the genetic markers were used to evaluate the responses of imputation accuracy on individual relationship, i.e. half sib vs. full sibs population.

b The half sib population was used to evaluate the responses of imputation accuracy on marker density. Two levels of marker density were examined. The high level marker density contained all the available markers (9990). The low density contained one fifth of the total available markers which are sampled evenly (choosing one out of every five adjacent markers).

Second, we examined the effect of markers density on imputation accuracy. The half sib family structure described above was used with two set of markers. One set contained all the available markers (9990 SNPs) with average LD of 0.137 and the other contained one fifth markers (1998 SNPs) with average LD of 0.092.Compared to BEAGLE and fastPHASE, iBLUP performed higher imputation accuracy at missing rate ranged from 60% to 80% in both cases (**Table 3**).

**Figure 2:**
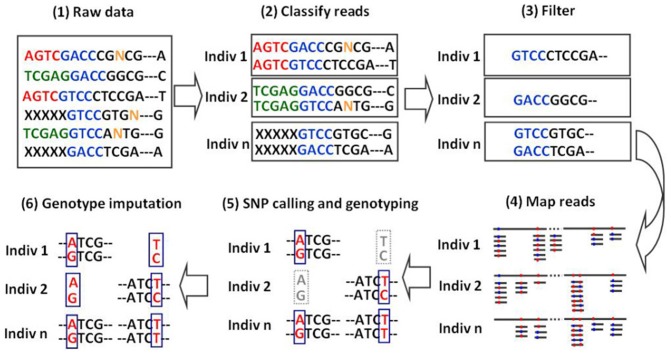
Diagram of iBLUP pipeline. (1) Blended raw data were generated from the same flow cell lane. (2) Raw data were assigned to individuals according to the barcode. (3) Assigned reads were filtered for high quality reads according to several rules, including trimming the barcode and the last low quality base etc. (4) Filtered reads were aligned with the reference sequence. (5) SNP calling and genotyping were done according to the mapping results. (6) Missing genotypes were imputed by the iBLUP algorithm.

We implemented the iBLUP algorithm in a publicly available pipeline also named iBLUP. The imputation step can be used independently for any genotype data, including the ones from DNA chips. The imputation step can also be used for raw sequencing data after four prior steps in iBLUP pipeline (**Figure 2**).

The iBLUP pipeline provides users the option to optimize the LD threshold to determine the extent of the LD block. We examined the imputation accuracy with LD thresholds of 0.05, 0.1 and 0.2 on the QTLMAS 15th dataset at missing rate of 30% (**Figure S2**). The analysis showed that an LD threshold of 0.1 would achieve the highest imputation accuracy. Interestingly, this threshold was also observed as the optimum value of LD threshold for the pig sequencing data. This observation might help narrow the optimization range for LD to reduce computing cost in other experiments.

We applied the iBLUP pipeline to sequencing data from a pig outbreed population for high-density SNP discovery and genotyping. The sequences were collected in one lane of a single flow-cell at 72-plex by a genome reducing and sequencing protocol (http://klab.sjtu.edu.cn/GGRS/). There were 36% of missing data among 403,928 SNPs called. The accuracy of imputation is 97% for iBLUP and 92% for BEAGLE. In order to make a comparison of imputation accuracy between iBLUP and BEAGLE, known genotypes were masked as missing at four other different rates: 50%, 60%, 70% and 80%. The imputation accuracy decreased with the increase of missing rates for both methods. The iBLUP method outperformed over BEAGLE at all range of missing rates for the real sequencing data (**Table 4**).

**Table 4:** Imputation accuracy of real pig sequencing data.

Method	36%	50%	60%	70%	80%
iBLIUP	0.97	0.97	0.96	0.96	0.95
BEAGLE	0.92	0.92	0.91	0.91	0.91

## Discussion

Missing genotype imputation is a critical process between sequencing and utilization for GWAS and genomic prediction [29]–[31]. Imputation accuracy relies on how well LD and IBD information are incorporated. IBD information is widely used in population and quantitative genetics. It is traditionally calculated from pedigree [32]. An alternative way to estimating IBD coefficients is from genetic markers [33]. This marker-based IBD more accurately specifies the actual difference between individuals and is superior to the pedigree kinship for genome-wide association studies [34]. The similar advantage was brought to genotype imputation in this study.

The accuracy improvement of iBLUP also relate to the optimization to determine the LD block. We demonstrate that it was critical to have an appropriate LD block for imputation. Too broad or too narrow LD blocks would lead to the information dilution (**Figure S2**). The best LD block size can be determined by the optimization process in iBLUP. The suggested LD threshold of 0.1 can be used to save computing time or a starting value of optimization.

The tolerance of iBLUP to high missing rate makes it possible to gain markers at high density. Take the pig data for example, haplotype blocks are about 10 kb within pig breed [35]. We need to identify markers that cover around 300,000 genomic locations at least for the GWAS or GS studies (one SNP per haplotype block). However, the commonly used pig DNA chip (PorcineSNP60) only contains 60,000 SNPs [36]. In the present pig sequencing experiment, we only use one lane of flow cell for 72-plex. After imputation of 36% of missing genotypes, we gained more than 403,928 SNPs, which has much better coverage than the commonly used chip.

One of the limitations of our proposed iBLUP is the computing speed for large sample size. When the sample size is medium (<300), the computing speed of iBLUP can compare with BEAGLE. Take the real 72 pig sequencing data (403,928 SNPs) for example, it takes about 20 minutes to perform imputation for both iBLUP and BEAGLE; 18 hours for fastPHASE. When the sample size is large, iBLUP will take more time than BEAGLE. To improve the computation speed, our iBLUP software can be run in parallel. Recently, factored spectrally transformed linear mixed models has been developed to improve the computing speed of genome-wide association studies [37], [38]. The idea can be applied in the iBLUP algorithm to improve the computing speed in the future.

A comprehensive package is provided at iBLUP website, including executable programs on multiple computing platforms (Linux and Windows) and demonstration data. The usage of iBLUP would boost imputation accuracy, especially for high missing genotypes and rare variants. Consequently it would lead to a better understanding the genetic architecture of complex traits in multiple organisms.

## Supporting Information

Figure S1 The scheme of sampling Individuals. The top panel (a) is the complete pedigree of the 15^th^QTLMAS workshop data [27]with 20 sires. Each Sire (S) mated with 10 Dams (D). Each dam produced 15 Progeny (P). All individuals are named randomly with sequential number. The first subscript indicates sire, the second indicates dam the third indicates progeny. The total numbers of individuals within each category are labeled on the fight column. The middle panel (b) keeps all the sires and dams. The difference (highlighted in red) is that each sire-dam family keeps only the first two progeny. This scheme has more families (all) and less progeny within family. As half sib is the major relationship among individuals, this scheme is named half sib scheme. The bottom panel (c) keeps the first 5 sires and their mates from panel a. Each sire-dam family keeps eleven progeny. This scheme has fewer families but more progeny within family. As full sib is the major relationship among individuals, this scheme is named full sib scheme.
(TIF)

Figure S2 Impact of linkage disequilibrium threshold. The Linkage Disequilibrium (LD) was calculated as the squared correlation coefficient. The adjacent markers with LD above the threshold were considered as a LD block to perform imputation. The evaluation was performed on subset of the 15^th^ QTLMAS common dataset by using the half-sib sampling scheme described in **Figure S1**. A total of 100 replications were conducted and the imputation accuracy is the average of 100 replications.
(TIF)

Table S1 Additional information of the 72 pigs that were sequenced.
(DOCX)

## References

[1] Price AL, Zaitlen NA, Reich D, Patterson N (2010) New approaches to population stratification in genome-wide association studies. Nat Rev Genet 11: 459–463. 20548291 10.1038/nrg2813 PMC2975875

[2] Mardis ER (2008) The impact of next-generation sequencing technology on genetics. Trends Genet 24: 133–141. 18262675 10.1016/j.tig.2007.12.007 PMC2680276

[3] Goddard ME, Hayes BJ (2009) Mapping genes for complex traits in domestic animals and their use in breeding programmes. Nat Rev Genet 10: 381–391. 19448663 10.1038/nrg2575

[4] Zuk O, Hechter E, Sunyaev SR, Lander ES (2011) The mystery of missing heritability: Genetic interactions create phantom heritability. Proc Natl Acad Sci U S A 109: 1193–1198. 10.1073/pnas.1119675109 PMC3268279 22223662

[5] Yang J, Benyamin B, McEvoy BP, Gordon S, Henders AK, et al (2010) Common SNPs explain a large proportion of the heritability for human height. Nat Genet 42: 565–569. 20562875 10.1038/ng.608 PMC3232052

[6] Maher B (2008) Personal genomes: The case of the missing heritability. Nature 456: 18–21. 18987709 10.1038/456018a

[7] Manolio TA, Collins FS, Cox NJ, Goldstein DB, Hindorff LA, et al (2009) Finding the missing heritability of complex diseases. Nature 461: 747–753. 19812666 10.1038/nature08494 PMC2831613

[8] Schork NJ, Murray SS, Frazer KA, Topol EJ (2009) Common vs. rare allele hypotheses for complex diseases. Curr Opin Genet Dev 19: 212–219. 19481926 10.1016/j.gde.2009.04.010 PMC2914559

[9] Church GM, Kieffer-Higgins S (1988) Multiplex DNA sequencing. Science 240: 185–188. 3353714 10.1126/science.3353714

[10] Elshire RJ, Glaubitz JC, Sun Q, Poland JA, Kawamoto K, et al (2011) A robust, simple genotyping-by-sequencing (GBS) approach for high diversity species. PloS one 6: e19379. 21573248 10.1371/journal.pone.0019379 PMC3087801

[11] Chen Q, Ma YF, Yang YM, Chen ZL, Liao RR, et al. (2013) Genotyping by Genome Reducing and Sequencing for Outbred Animals. PLoS ONE: journal.pone.0067500. 10.1371/journal.pone.0067500 PMC3715491 23874423

[12] Li Y, Willer C, Sanna S, Abecasis G (2009) Genotype imputation. Annu Rev Genomics Hum Genet 10: 387–406. 19715440 10.1146/annurev.genom.9.081307.164242 PMC2925172

[13] Marchini J, Howie B (2010) Genotype imputation for genome-wide association studies. Nat Rev Genet 11: 499–511. 20517342 10.1038/nrg2796

[14] Browning SR, Browning BL (2007) Rapid and accurate haplotype phasing and missing-data inference for whole-genome association studies by use of localized haplotype clustering. The American Journal of Human Genetics 81: 1084–1097. 17924348 10.1086/521987 PMC2265661

[15] Browning BL, Browning SR (2009) A unified approach to genotype imputation and haplotype-phase inference for large data sets of trios and unrelated individuals. The American Journal of Human Genetics 84: 210–223. 19200528 10.1016/j.ajhg.2009.01.005 PMC2668004

[16] Purcell S, Neale B, Todd-Brown K, Thomas L, Ferreira MA, et al (2007) PLINK: a tool set for whole-genome association and population-based linkage analyses. Am J Hum Genet 81: 559–575. 17701901 10.1086/519795 PMC1950838

[17] Lin DY, Hu Y, Huang BE (2008) Simple and efficient analysis of disease association with missing genotype data. Am J Hum Genet 82: 444–452. 18252224 10.1016/j.ajhg.2007.11.004 PMC2427170

[18] Nicolae DL (2006) Testing untyped alleles (TUNA)-applications to genome-wide association studies. Genet Epidemiol 30: 718–727. 16986160 10.1002/gepi.20182

[19] Howie BN, Donnelly P, Marchini J (2009) A flexible and accurate genotype imputation method for the next generation of genome-wide association studies. PLoS Genet 5: e1000529. 19543373 10.1371/journal.pgen.1000529 PMC2689936

[20] Scheet P, Stephens M (2006) A fast and flexible statistical model for large-scale population genotype data: applications to inferring missing genotypes and haplotypic phase. Am J Hum Genet 78: 629–644. 16532393 10.1086/502802 PMC1424677

[21] Gengler N, Mayeres P, Szydlowski M (2007) A simple method to approximate gene content in large pedigree populations: application to the myostatin gene in dual-purpose Belgian Blue cattle. Animal 1: 21–28. 22444206 10.1017/S1751731107392628

[22] Mulder HA, Meuwissen TH, Calus MP, Veerkamp RF (2010) The effect of missing marker genotypes on the accuracy of gene-assisted breeding value estimation: a comparison of methods. Animal 4: 9–19. 22443613 10.1017/S1751731109990838

[23] Chen Q, Ma Y, Yang Y, Chen Z, Liao R, et al (2013) Genotyping by genome reducing and sequencing for outbred animals. PLoS One 8: e67500. 23874423 10.1371/journal.pone.0067500 PMC3715491

[24] Xu S (2013) Mapping quantitative trait loci by controlling polygenic background effects. Genetics 195: 1209–1222. 24077303 10.1534/genetics.113.157032 PMC3832267

[25] Li H, Durbin R (2009) Fast and accurate short read alignment with Burrows-Wheeler transform. Bioinformatics 25: 1754–1760. 19451168 10.1093/bioinformatics/btp324 PMC2705234

[26] Li H, Handsaker B, Wysoker A, Fennell T, Ruan J, et al (2009) The sequence alignment/map format and SAMtools. Bioinformatics 25: 2078–2079. 19505943 10.1093/bioinformatics/btp352 PMC2723002

[27] Elsen JM, Tesseydre S, Filangi O, Le Roy P, Demeure O (2012) XVth QTLMAS: simulated dataset. BMC Proc 6 Suppl 2S1. 10.1186/1753-6561-6-S2-S1 PMC3363151 22640408

[28] Marchini J, Howie B (2010) Genotype imputation for genome-wide association studies. Nature Reviews Genetics 11: 499–511. 10.1038/nrg2796 20517342

[29] Hayes BJ, Bowman PJ, Chamberlain AJ, Goddard ME (2009) Invited review: Genomic selection in dairy cattle: progress and challenges. J Dairy Sci 92: 433–443. 19164653 10.3168/jds.2008-1646

[30] Andolfatto P, Davison D, Erezyilmaz D, Hu TT, Mast J, et al (2011) Multiplexed shotgun genotyping for rapid and efficient genetic mapping. Genome Res 21: 610–617. 21233398 10.1101/gr.115402.110 PMC3065708

[31] Nielsen R, Paul JS, Albrechtsen A, Song YS (2011) Genotype and SNP calling from next-generation sequencing data. Nat Rev Genet 12: 443–451. 21587300 10.1038/nrg2986 PMC3593722

[32] Wright SI (1922) Coefficient of inbreeding and relationship. The American Naturalist 56: 330–338.

[33] Frazer KA, Ballinger DG, Cox DR, Hinds DA, Stuve LL, et al (2007) A second generation human haplotype map of over 3.1 million SNPs. Nature 449: 851–861. 17943122 10.1038/nature06258 PMC2689609

[34] Hayes BJ, Visscher PM, Goddard ME (2009) Increased accuracy of artificial selection by using the realized relationship matrix. Genet Res 91: 47–60. 10.1017/S0016672308009981 19220931

[35] Amaral AJ, Megens HJ, Crooijmans RP, Heuven HC, Groenen MA (2008) Linkage disequilibrium decay and haplotype block structure in the pig. Genetics 179: 569–579. 18493072 10.1534/genetics.107.084277 PMC2390633

[36] Vingborg RK, Gregersen VR, Zhan B, Panitz F, Hoj A, et al (2009) A robust linkage map of the porcine autosomes based on gene-associated SNPs. BMC Genomics 10: 134. 19327136 10.1186/1471-2164-10-134 PMC2674067

[37] Lippert C, Listgarten J, Liu Y, Kadie CM, Davidson RI, et al (2011) FaST linear mixed models for genome-wide association studies. Nat Methods 8: 833–835. 21892150 10.1038/nmeth.1681

[38] Listgarten J, Lippert C, Kadie CM, Davidson RI, Eskin E, et al (2012) Improved linear mixed models for genome-wide association studies. Nat Methods 9: 525–526. 22669648 10.1038/nmeth.2037 PMC3597090

