# Supplementary figures and images for: A New Genotype Imputation Method with Tolerance to High Missing Rate and Rare Variants
Source: PLoS One. 2014 Jun 27;9(6):e101025. doi: 10.1371/journal.pone.0101025 (PMC4074155; doi:10.1371/journal.pone.0101025)

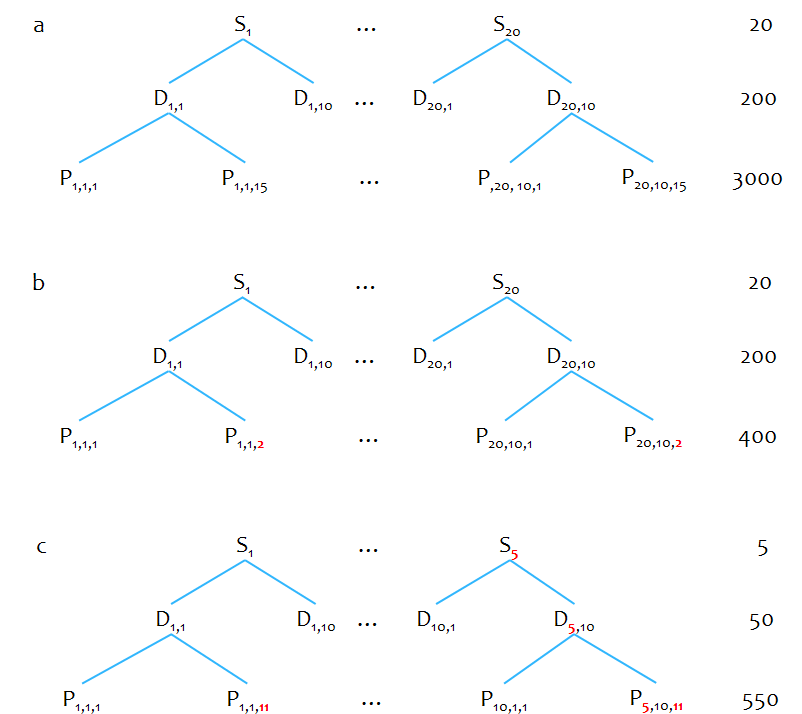

Supplement: Figure S1 — The scheme of sampling Individuals. The top panel (a) is the complete pedigree of the 15thQTLMAS workshop data [27]with 20 sires. Each Sire (S) mated with 10 Dams (D). Each dam produced 15 Progeny (P). All individuals are named randomly with sequential number. The first subscript indicates sire, the second indicates dam the third indicates progeny. The total numbers of individuals within each category are labeled on the fight column. The middle panel (b) keeps all the sires and dams. The difference (highlighted in red) is that each sire-dam family keeps only the first two progeny. This scheme has more families (all) and less progeny within family. As half sib is the major relationship among individuals, this scheme is named half sib scheme. The bottom panel (c) keeps the first 5 sires and their mates from panel a. Each sire-dam family keeps eleven progeny. This scheme has fewer families but more progeny within family. As full sib is the major relationship among individuals, this scheme is named full sib scheme. (TIF) [file pone.0101025.s001.tif]
