# Supplementary material for: A New Genotype Imputation Method with Tolerance to High Missing Rate and Rare Variants
Source: PLoS One. 2014 Jun 27;9(6):e101025. doi: 10.1371/journal.pone.0101025 (PMC4074155; doi:10.1371/journal.pone.0101025)
Supplement: Table S1 — Additional information of the 72 pigs that were sequenced. (DOCX) [file pone.0101025.s003.docx]

| Barcode | Breed | Gender | Farm | Adapter-barcode | Adapter-barcode base number |
| --- | --- | --- | --- | --- | --- |
| AACT | Landrance | Female | Shanghai Xiangxin Livestock Ltd. Co. | ACACTCTTTCCCTACACGACGCTCTTCCGATCTAACT | 37 |
| CAGA | Landrance | Female | Shanghai Xiangxin Livestock Ltd. Co. | ACACTCTTTCCCTACACGACGCTCTTCCGATCTCAGA | 37 |
| CGAT | Landrance | Female | Shanghai Xiangxin Livestock Ltd. Co. | ACACTCTTTCCCTACACGACGCTCTTCCGATCTCGAT | 37 |
| GATC | Landrance | Female | Shanghai Xiangxin Livestock Ltd. Co. | ACACTCTTTCCCTACACGACGCTCTTCCGATCTGATC | 37 |
| GCGT | Landrance | Female | Shanghai Xiangxin Livestock Ltd. Co. | ACACTCTTTCCCTACACGACGCTCTTCCGATCTGCGT | 37 |
| GTAA | Landrance | Female | Shanghai Xiangxin Livestock Ltd. Co. | ACACTCTTTCCCTACACGACGCTCTTCCGATCTGTAA | 37 |
| TCAC | Landrance | Female | Shanghai Xiangxin Livestock Ltd. Co. | ACACTCTTTCCCTACACGACGCTCTTCCGATCTTCAC | 37 |
| TGCA | Landrance | Female | Shanghai Xiangxin Livestock Ltd. Co. | ACACTCTTTCCCTACACGACGCTCTTCCGATCTTGCA | 37 |
| ACAAA | Landrance | Female | Shanghai Xiangxin Livestock Ltd. Co. | ACACTCTTTCCCTACACGACGCTCTTCCGATCTACAAA | 38 |
| AGCCC | Landrance | Female | Shanghai Xiangxin Livestock Ltd. Co. | ACACTCTTTCCCTACACGACGCTCTTCCGATCTAGCCC | 38 |
| AGGAT | Landrance | Female | Shanghai Xiangxin Livestock Ltd. Co. | ACACTCTTTCCCTACACGACGCTCTTCCGATCTAGGAT | 38 |
| CATCT | Landrance | Female | Shanghai Xiangxin Livestock Ltd. Co. | ACACTCTTTCCCTACACGACGCTCTTCCGATCTCATCT | 38 |
| CCTAC | Landrance | Female | Shanghai Xiangxin Livestock Ltd. Co. | ACACTCTTTCCCTACACGACGCTCTTCCGATCTCCTAC | 38 |
| CTGTA | Landrance | Female | Shanghai Xiangxin Livestock Ltd. Co. | ACACTCTTTCCCTACACGACGCTCTTCCGATCTCTGTA | 38 |
| GAGGA | Landrance | Female | Shanghai Xiangxin Livestock Ltd. Co. | ACACTCTTTCCCTACACGACGCTCTTCCGATCTGAGGA | 38 |
| GGAAC | Landrance | Female | Shanghai Xiangxin Livestock Ltd. Co. | ACACTCTTTCCCTACACGACGCTCTTCCGATCTGGAAC | 38 |
| GGTGT | Landrance | Female | Shanghai Xiangxin Livestock Ltd. Co. | ACACTCTTTCCCTACACGACGCTCTTCCGATCTGGTGT | 38 |
| GTATT | Landrance | Female | Shanghai Xiangxin Livestock Ltd. Co. | ACACTCTTTCCCTACACGACGCTCTTCCGATCTGTATT | 38 |
| GTCAA | Landrance | Female | Shanghai Xiangxin Livestock Ltd. Co. | ACACTCTTTCCCTACACGACGCTCTTCCGATCTGTCAA | 38 |
| TAATA | Landrance | Female | Shanghai Xiangxin Livestock Ltd. Co. | ACACTCTTTCCCTACACGACGCTCTTCCGATCTTAATA | 38 |
| TACAT | Landrance | Female | Shanghai Xiangxin Livestock Ltd. Co. | ACACTCTTTCCCTACACGACGCTCTTCCGATCTTACAT | 38 |
| TCACC | Landrance | Female | Shanghai Xiangxin Livestock Ltd. Co. | ACACTCTTTCCCTACACGACGCTCTTCCGATCTTCACC | 38 |
| TCGTT | Landrance | Female | Shanghai Xiangxin Livestock Ltd. Co. | ACACTCTTTCCCTACACGACGCTCTTCCGATCTTCGTT | 38 |
| TGCGA | Landrance | Female | Shanghai Xiangxin Livestock Ltd. Co. | ACACTCTTTCCCTACACGACGCTCTTCCGATCTTGCGA | 38 |
| TTCTC | Landrance | Female | Shanghai Xiangxin Livestock Ltd. Co. | ACACTCTTTCCCTACACGACGCTCTTCCGATCTTTCTC | 38 |
| ACCTAA | Landrance | Female | Shanghai Xiangxin Livestock Ltd. Co. | ACACTCTTTCCCTACACGACGCTCTTCCGATCTACCTAA | 39 |
| ATATGT | Landrance | Female | Shanghai Xiangxin Livestock Ltd. Co. | ACACTCTTTCCCTACACGACGCTCTTCCGATCTATATGT | 39 |
| CCAGCT | Landrance | Female | Shanghai Xiangxin Livestock Ltd. Co. | ACACTCTTTCCCTACACGACGCTCTTCCGATCTCCAGCT | 39 |
| GAGATA | Landrance | Female | Shanghai Xiangxin Livestock Ltd. Co. | ACACTCTTTCCCTACACGACGCTCTTCCGATCTGAGATA | 39 |
| GCCAGT | Landrance | Female | Shanghai Xiangxin Livestock Ltd. Co. | ACACTCTTTCCCTACACGACGCTCTTCCGATCTGCCAGT | 39 |
| GCTCTA | Landrance | Male | Shanghai Xiangxin Livestock Ltd. Co. | ACACTCTTTCCCTACACGACGCTCTTCCGATCTGCTCTA | 39 |
| GGTTGT | Landrance | Male | Shanghai Xiangxin Livestock Ltd. Co. | ACACTCTTTCCCTACACGACGCTCTTCCGATCTGGTTGT | 39 |
| TAACGA | Landrance | Male | Shanghai Xiangxin Livestock Ltd. Co. | ACACTCTTTCCCTACACGACGCTCTTCCGATCTTAACGA | 39 |
| TGGCTA | Landrance | Male | Shanghai Xiangxin Livestock Ltd. Co. | ACACTCTTTCCCTACACGACGCTCTTCCGATCTTGGCTA | 39 |
| TTCAGA | Landrance | Male | Shanghai Xiangxin Livestock Ltd. Co. | ACACTCTTTCCCTACACGACGCTCTTCCGATCTTTCAGA | 39 |
| AACGCCT | Landrance | Male | Shanghai Xiangxin Livestock Ltd. Co. | ACACTCTTTCCCTACACGACGCTCTTCCGATCTAACGCCT | 40 |
| AATATGC | Large white | Female | Shanghai Xiangxin Livestock Ltd. Co. | ACACTCTTTCCCTACACGACGCTCTTCCGATCTAATATGC | 40 |
| ATGAAAC | Large white | Female | Shanghai Xiangxin Livestock Ltd. Co. | ACACTCTTTCCCTACACGACGCTCTTCCGATCTATGAAAC | 40 |
| CGGTAGA | Large white | Female | Shanghai Xiangxin Livestock Ltd. Co. | ACACTCTTTCCCTACACGACGCTCTTCCGATCTCGGTAGA | 40 |
| GTCGATT | Large white | Female | Shanghai Xiangxin Livestock Ltd. Co. | ACACTCTTTCCCTACACGACGCTCTTCCGATCTGTCGATT | 40 |
| TCGAAGA | Large white | Female | Shanghai Xiangxin Livestock Ltd. Co. | ACACTCTTTCCCTACACGACGCTCTTCCGATCTTCGAAGA | 40 |
| AAAAGTT | Large white | Female | Shanghai Xiangxin Livestock Ltd. Co. | ACACTCTTTCCCTACACGACGCTCTTCCGATCTAAAAGTT | 40 |
| AACCGAGA | Large white | Female | Shanghai Xiangxin Livestock Ltd. Co. | ACACTCTTTCCCTACACGACGCTCTTCCGATCTAACCGAGA | 41 |
| ACGACTAC | Large white | Female | Shanghai Xiangxin Livestock Ltd. Co. | ACACTCTTTCCCTACACGACGCTCTTCCGATCTACGACTAC | 41 |
| ACGTGTT | Large white | Female | Shanghai Xiangxin Livestock Ltd. Co. | ACACTCTTTCCCTACACGACGCTCTTCCGATCTACGTGTT | 40 |
| AGGC | Large white | Female | Shanghai Xiangxin Livestock Ltd. Co. | ACACTCTTTCCCTACACGACGCTCTTCCGATCTAGGC | 37 |
| AGTGGA | Large white | Female | Shanghai Xiangxin Livestock Ltd. Co. | ACACTCTTTCCCTACACGACGCTCTTCCGATCTAGTGGA | 39 |
| ATGCCT | Large white | Female | Shanghai Xiangxin Livestock Ltd. Co. | ACACTCTTTCCCTACACGACGCTCTTCCGATCTATGCCT | 39 |
| ATTAATT | Large white | Female | Shanghai Xiangxin Livestock Ltd. Co. | ACACTCTTTCCCTACACGACGCTCTTCCGATCTATTAATT | 40 |
| ATTGA | Large white | Female | Shanghai Xiangxin Livestock Ltd. Co. | ACACTCTTTCCCTACACGACGCTCTTCCGATCTATTGA | 38 |
| CATCGT | Large white | Female | Shanghai Xiangxin Livestock Ltd. Co. | ACACTCTTTCCCTACACGACGCTCTTCCGATCTCATCGT | 39 |
| CCACAA | Large white | Female | Shanghai Xiangxin Livestock Ltd. Co. | ACACTCTTTCCCTACACGACGCTCTTCCGATCTCCACAA | 39 |
| CCGGATAT | Large white | Female | Shanghai Xiangxin Livestock Ltd. Co. | ACACTCTTTCCCTACACGACGCTCTTCCGATCTCCGGATAT | 41 |
| CGCGGAGA | Large white | Female | Shanghai Xiangxin Livestock Ltd. Co. | ACACTCTTTCCCTACACGACGCTCTTCCGATCTCGCGGAGA | 41 |
| CGCTGAT | Large white | Female | Shanghai Xiangxin Livestock Ltd. Co. | ACACTCTTTCCCTACACGACGCTCTTCCGATCTCGCTGAT | 40 |
| CGCTT | Large white | Female | Shanghai Xiangxin Livestock Ltd. Co. | ACACTCTTTCCCTACACGACGCTCTTCCGATCTCGCTT | 38 |
| CGTGTGGT | Large white | Female | Shanghai Xiangxin Livestock Ltd. Co. | ACACTCTTTCCCTACACGACGCTCTTCCGATCTCGTGTGGT | 41 |
| CTAGC | Large white | Female | Shanghai Xiangxin Livestock Ltd. Co. | ACACTCTTTCCCTACACGACGCTCTTCCGATCTCTAGC | 38 |
| CTATTA | Large white | Female | Shanghai Xiangxin Livestock Ltd. Co. | ACACTCTTTCCCTACACGACGCTCTTCCGATCTCTATTA | 39 |
| CTCC | Large white | Female | Shanghai Xiangxin Livestock Ltd. Co. | ACACTCTTTCCCTACACGACGCTCTTCCGATCTCTCC | 37 |
| CTTGCTT | Large white | Female | Shanghai Xiangxin Livestock Ltd. Co. | ACACTCTTTCCCTACACGACGCTCTTCCGATCTCTTGCTT | 40 |
| GAACTTC | Large white | Female | Shanghai Xiangxin Livestock Ltd. Co. | ACACTCTTTCCCTACACGACGCTCTTCCGATCTGAACTTC | 40 |
| GCTGTGGA | Large white | Female | Shanghai Xiangxin Livestock Ltd. Co. | ACACTCTTTCCCTACACGACGCTCTTCCGATCTGCTGTGGA | 41 |
| GGACCTA | Large white | Female | Shanghai Xiangxin Livestock Ltd. Co. | ACACTCTTTCCCTACACGACGCTCTTCCGATCTGGACCTA | 40 |
| GGATTGGT | Large white | Female | Shanghai Xiangxin Livestock Ltd. Co. | ACACTCTTTCCCTACACGACGCTCTTCCGATCTGGATTGGT | 41 |
| GTTGAA | Large white | Female | Shanghai Xiangxin Livestock Ltd. Co. | ACACTCTTTCCCTACACGACGCTCTTCCGATCTGTTGAA | 39 |
| TAGGAA | Large white | Male | Shanghai Xiangxin Livestock Ltd. Co. | ACACTCTTTCCCTACACGACGCTCTTCCGATCTTAGGAA | 39 |
| TAGGCCAT | Large white | Male | Shanghai Xiangxin Livestock Ltd. Co. | ACACTCTTTCCCTACACGACGCTCTTCCGATCTTAGGCCAT | 41 |
| TATTTTT | Large white | Male | Shanghai Xiangxin Livestock Ltd. Co. | ACACTCTTTCCCTACACGACGCTCTTCCGATCTTATTTTT | 40 |
| TCTCAGTC | Large white | Male | Shanghai Xiangxin Livestock Ltd. Co. | ACACTCTTTCCCTACACGACGCTCTTCCGATCTTCTCAGTC | 41 |
| TCTGTGA | Large white | Male | Shanghai Xiangxin Livestock Ltd. Co. | ACACTCTTTCCCTACACGACGCTCTTCCGATCTTCTGTGA | 40 |
| TTCCTGGA | Large white | Male | Shanghai Xiangxin Livestock Ltd. Co. | ACACTCTTTCCCTACACGACGCTCTTCCGATCTTTCCTGGA | 41 |
